# Serum Insulin-like Growth Factor-Binding Protein-2 as a Prognostic Factor for COVID-19 Severity

**DOI:** 10.3390/biomedicines12010125

**Published:** 2024-01-08

**Authors:** Patricia Mester, Ulrich Räth, Stephan Schmid, Pablo Amend, Dennis Keller, Sabrina Krautbauer, Sofiia Bondarenko, Martina Müller, Christa Buechler, Vlad Pavel

**Affiliations:** 1 Department of Internal Medicine I, Gastroenterology, Hepatology, Endocrinology, Rheumatology, and Infectious Diseases, University Hospital Regensburg, 93053 Regensburg, Germany; patricia.mester@klinik.uni-regensburg.de (P.M.); ulrich.raeth@stud.uni-regensburg.de (U.R.); stephan.schmid@klinik.uni-regensburg.de (S.S.); pablo.amend@stud.uni-regensburg.de (P.A.); dennis.keller@stud.uni-regensburg.de (D.K.); martina.mueller-schilling@klinik.uni-regensburg.de (M.M.); vlad.pavel@klinik.uni-regensburg.de (V.P.); 2 Institute of Clinical Chemistry and Laboratory Medicine, University Hospital Regensburg, 93053 Regensburg, Germany; sabrina.krautbauer@klinik.uni-regensburg.de (S.K.); sofiia.bondarenko@klinik.uni-regensburg.de (S.B.)

**Keywords:** COVID-19, prognosis, IGFBP-2, intensive care, mortality, procalcitonin

## Abstract

Insulin-like growth factor-binding protein (IGFBP)-2 is a regulator of anabolic pathways, which become inactivated in severe illness. Here, we measured the serum IGFBP-2 levels of COVID-19 patients with moderate and severe disease as well as healthy controls to identify the associations of serum IGFBP-2 levels with disease severity. Patients with severe COVID-19 had higher serum IGFBP-2 levels than those with moderate disease and healthy controls, who had similar levels. Non-survivors of COVID-19 tended to have elevated serum IGFBP-2 levels compared to survivors. Increased serum IGFBP-2 levels were observed in patients requiring dialysis and vasopressor therapy. Serum IGFBP-2 was positively correlated with procalcitonin in both patient groups. Bacterial co-infection in severe COVID-19 patients did not influence serum IGFBP-2 levels. Patients with liver cirrhosis and obesity, showing increased and decreased serum IGFBP-2 levels, respectively, were excluded from the study. The present analysis showed that higher serum IGFBP-2 levels are associated with increased disease severity in COVID-19 patients. The similarity in serum IGFBP-2 levels between patients with moderate COVID-19 and healthy controls suggests that elevated IGFBP-2 is associated with critical illness rather than SARS-CoV-2 infection itself.

## 1. Introduction

Critical illness is a hypercatabolic state characterized by low levels of the anabolic hormone insulin-like growth factor 1 (IGF1) [1,2]. IGF1 binds to the IGF1 receptor and activates AKT, which has important roles in cell growth, proliferation, and metabolism [2,3]. IGF1 increases the cellular uptake of amino acids and reduces protein breakdown in muscle cells [2]. IGF1 bioactivity is regulated by insulin-like growth factor-binding proteins (IGFBPs) [1,2]. Most of the circulating IGF1 is bound to IGFBP-3, and the binding of IGFBP-3 to IGF1 increases its half-life. Systemic IGFBP-3 levels are reduced in severe illness [4,5], and this may interfere with IGF-regulated pathways.

IGFBP-2 is another IGF-binding protein that has been shown to negatively affect the bioactivity of IGFs [6]. There is evidence to suggest that IGF1 increases IGFBP-2 expression in the kidneys and lungs [7]; however, whether systemic levels of IGFBP-2 are related to IGF1 levels has not been conclusively established.

IGFBP-2 exhibits IGF-dependent and IGF-independent activities, and the binding of IGFBP-2 to integrins induces phosphatase and tensin homolog (PTEN) phosphorylation [2,8]. PTEN is a key negative regulator of AKT and is inactivated by phosphorylation [9]. The IGFBP2-mediated inactivation of PTEN, and subsequent activation of AKT, stimulates cell growth [2]. Because of its role in cell proliferation, IGFBP-2 has been studied mainly in different types of cancer. High expression of IGFBP-2 in cancer tissues has been associated with a worse outcome [10]. Current evidence suggests that serum IGFBP-2 may become a diagnostic marker and therapeutic target for patients with various malignancies [11].

The expression and circulating levels of IGFBP-2 in critically ill patients have not been thoroughly investigated. Endotoxins are very potent pro-inflammatory substances from Gram-negative bacteria that induce acute non-specific inflammation [12]. Endotoxin injection in healthy volunteers resulted in a decrease in IGF1 and higher levels of IGFBP-2 in the blood [13]. The proinflammatory cytokine tumor necrosis factor strongly induced IGFBP-2 in lung alveolar epithelial cells [14]. Estrogens have been extensively studied for their potential benefits in the treatment of sepsis. However, bioavailable estradiol levels were found to be elevated in sepsis patients, particularly in those who did not survive [15,16]. Estrogens have been demonstrated to induce IGFBP-2 in various cells and organs, including the liver [7]. Estrogens were also described to reduce circulating IGFBP-2 levels, [17] and further studies have to evaluate the effect of estrogen on serum IGFBP-2 levels.

In accordance with the upregulation of IGFBP-2 by inflammatory factors, higher levels were observed in critical illness. On admission to hospital, serum IGFBP-2 levels in critically ill patients were above the normal range and did not change during 30 days of follow-up [4]. A second study reported almost two times higher levels of IGFBP-2 in the serum of severely ill patients compared to healthy controls [5].

Severe acute respiratory syndrome coronavirus type 2 (SARS-CoV-2) belongs to the family Coronaviridae. The SARS-CoV-2 genome is a single-stranded, positive-sense, 26–32 kb RNA with a nucleocapsid protein, which is packaged in an envelope and is responsible for viral replication in host cells. The viral envelope is composed of three types of structural proteins: membrane, spike, and envelope protein. The trimeric spike protein plays a key role in viral entry into host cells [18]. The spike protein binds to angiotensin-converting enzyme 2 receptor, which is expressed on the cellular surfaces of most human cells [19].

The lungs are the first organ infected by the virus, but other vital organs such as the liver, heart, kidneys, brain, and blood vessels can be also infected in COVID-19 patients [19]. SARS-CoV-2 infection of the lung tissues can induce a cytokine storm, the infiltration of immune cells to tissues, and coagulation abnormalities, which may secondarily lead to multi-organ dysfunction and death [20]. Circulating levels of various cytokines such as interleukin-6 and tumor necrosis factor are increased in severe COVID-19 [21], and associations with disease severity and outcome have been described [22,23].

Coronavirus disease 2019 (COVID-19) has a range of clinical symptoms, from asymptomatic infections to critical illness presenting with multiorgan failure [24]. SARS-CoV-2 infection causes severe illness in about 20% of patients, and about 5% of these patients require intensive care treatment [25]. The risk factors for severe COVID-19 are numerous and include older age, male gender, obesity, and metabolic diseases such as type 2 diabetes [26]. Acute respiratory distress syndrome (ARDS) is a very severe manifestation of SARS-CoV-2 infection, and about 40% of these patients have a worse outcome [27,28,29].

Serum IGF1 levels were found to be reduced in severe COVID-19 patients, in contrast to patients with mild disease [30]. Higher IGF1 levels were associated with better survival of patients with severe COVID-19 [31]. However, it has also been reported that patients with severe COVID-19 and healthy controls have comparable serum levels of IGF1 [32]. IGFBP-2 levels in COVID-19 patients have been described in one study, and plasma IGFBP-2 was found to be elevated in hospitalized COVID-19 patients compared to healthy controls in a proteomic analysis [33].

The identification of biomarkers of COVID-19 severity will help to elucidate disease mechanisms and guide the management of these critically ill patients. The aim of this study was to analyze the associations of serum IGFBP-2 levels with COVID-19 severity, and therefore, serum IGFBP-2 levels were determined in healthy controls and patients with moderate and severe COVID-19 disease.

## 2. Materials and Methods

### 2.1. Study Cohort

Serum samples from patients aged 18 and older were collected during their hospital stay from 16 April 2020 to 14 June 2021. Blood probes were collected mostly within 72 h after hospitalization. In a few cases, due to delays in receiving patient consent, the blood samples were collected a couple of days later. All personnel responsible for handling blood samples underwent comprehensive safety training. They were required to wear face masks with filtering facepiece (FFP) 2, lab coats, and gloves. Blood was handled in biosafety level 2 laboratories using standard precautions. SARS-CoV-2 infection was confirmed using polymerase chain reaction. As 57 patients presented with dyspnea, tachycardia, fever, and fatigue and fulfilled the systemic inflammatory response syndrome (SIRS) criteria, they were included in the moderate COVID-19 group [34]. Of note, our SIRS group corresponded clinically with moderate COVID-19 according to the National Institutes of Health (NIH) classification of COVID-19 severity [35]. These patients were hospitalized for close monitoring but did not need admission to the intensive care unit. Sixty patients were treated in the intensive care unit as most of them developed ARDS and septic shock. Our severe COVID-19 group corresponded with critical illness according to NIH classification of COVID-19 severity [35,36,37,38].

The partial arterial pressure of oxygen (PaO^2^, mmHg)/fraction of inspired oxygen (FiO^2^) index, also known as the Horowitz index, was calculated for all patients with COVID-19 treated on the intensive care unit and receiving invasive ventilation. An index below 200 mmHg was defined as ARDS. The median Horowitz index was calculated to be 147 (range 21–302) mmHg.

The COVID-19 patients received treatment in accordance with the current COVID-19 guidelines, as approved by the European Medicines Agency and the German Federal Joint Committee. In that period, the only approved drugs for the treatment of COVID-19 in Germany were remdesivir and dexamethasone. In the severe group, 57 patients were treated with dexamethasone, and 34 patients were treated with dexamethasone in the moderate COVID-19 cohort. Remdesivir was given to all but 16 patients.

To prevent thrombosis, all patients received either low-molecular-weight heparin or unfractionated heparin. Vaccination against SARS-CoV-2 began in Germany on 26 December 2020. It appears that most of our patients have not yet been vaccinated.

Drugs targeting cytokine release syndrome in COVID-19, like tocilizumab, baricitinib, and sotrovimab, were not approved in Germany at the time, and therefore, were not administrated to any patient.

The control group consisted of 23 individuals (12 males and 11 females), aged between 24 and 67 years, with a similar age and gender distribution to the COVID-19 patients.

### 2.2. IGFBP-2 ELISA

The human IGFBP-2 DuoSet ELISA (R&D Systems; Wiesbaden, Nordenstadt, Germany) was used according to the manufacturer’s instructions, with serum diluted in a ratio of 1:100 for analysis. Serum levels of IGFBP-2 were measured in duplicate and, for calculations, the mean IGFBP-2 values were used.

### 2.3. Statistical Analysis

Serum levels of IGFBP-2 are shown as box plots, and outliers are represented by single asterisks or circles. The data in the tables show the median, minimum, and maximum IGFBP-2 serum values. The statistical tests used were (1) a non-parametric Mann–Whitney U test, (2) a non-parametric Kruskal–Wallis test, and (3) Spearman’s correlation. The Chi-square test was used for calculations of categorical variables. IBM SPSS Statistics 26.0 was used for all statistical tests. A value of *p* < 0.05 was considered significant.

## 3. Results

### 3.1. Serum IGFBP-2 Levels of Controls and COVID-19 Patients

This study examined the serum of 117 COVID-19 patients, divided into 57 with moderate and 60 with severe disease. Patients with severe disease, who needed intensive care, had higher body mass indexes (BMIs), elevated levels of C-reactive protein (CRP), procalcitonin, ferritin, and lactate dehydrogenase (LDH), and increased counts of neutrophils, monocytes, and immature granulocytes compared to those with moderate disease. Both groups had similar gender distribution, ages, and alkaline phosphatase (AP) and interleukin-6 (IL-6) levels (Table 1).

Serum IGFBP-2 levels between the 43 female and the 74 male patients were similar (*p* = 0.125). IGFBP-2 correlated with the patients’ ages (r = 0.605, *p* < 0.001). Associations with age were not observed in the controls (r = −0.152, *p* = 0.522). In the control group, women had higher serum IGFBP-2 levels than men (*p* = 0.042).

**Figure 1:**
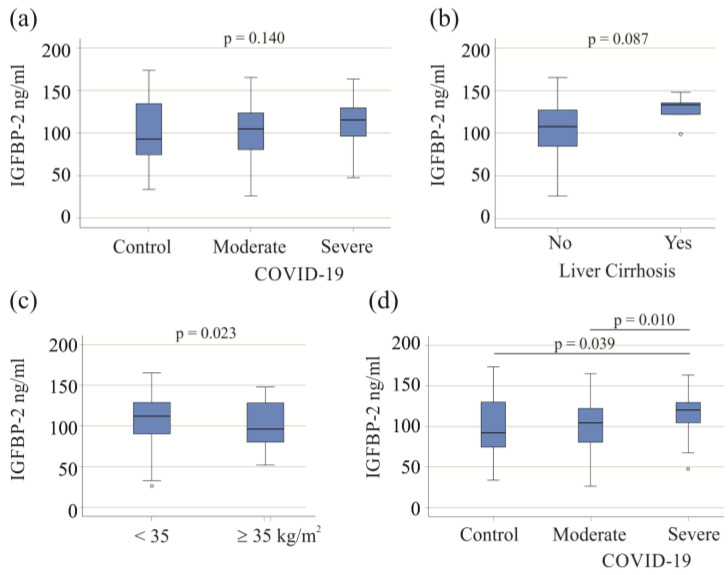
Serum IGFBP-2 levels of COVID-19 patients. (**a**) Serum IGFBP-2 levels of controls and patients with moderate and severe COVID-19; (**b**) serum IGFBP-2 levels of COVID-19 patients with (5 patients) and without liver cirrhosis; (**c**) serum IGFBP-2 levels of severe COVID-19 patients with a BMI < or ≥35 kg/m^2^; (**d**) serum IGFBP-2 levels of controls and patients with moderate and severe COVID-19 after excluding patients with liver cirrhosis and patients with adiposity.

The 57 moderate COVID-19 patients exhibited IGFBP-2 levels similar to those of the 60 severe patients and 23 healthy controls (*p* = 0.140; Figure 1a). In the study cohort, five patients with liver cirrhosis, a condition known to affect plasma protein levels [39], did not show significant changes in serum IGFBP-2 levels (*p* = 0.087; Figure 1b). There was a negative correlation between serum IGFBP-2 and BMI (r = −0.271, *p* = 0.015). Among the COVID-19 patients, 18 had a BMI ≥ 35 kg/m^2^, with 16 in the severe COVID-19 category. These 16 obese patients had lower serum IGFBP-2 levels compared to severely ill patients with a BMI < 35 kg/m^2^ (*p* = 0.023; Figure 1c).

Liver cirrhosis and adiposity affect serum levels of IGFBP-2 (Figure 1b,c), and the exclusion of these patients showed that the serum IGFBP-2 levels of patients with severe COVID-19 were higher in comparison to those of patients with moderate disease and healthy controls. These two latter groups had similar levels of serum IGFBP-2 (Figure 1d). For further analysis, patients with liver cirrhosis and adiposity were excluded.

Serum from our patients with severe COVID-19 was collected 4 (1–10) days after admission to the intensive care unit. IGFBP-2 (*p* = 0.679), CRP (*p* = 0.641), procalcitonin (*p* = 0.981), and IL-6 (*p* = 0.669) were not related to the day of blood collection.

### 3.2. IGFBP-2 in Relation to Interventions and Vasopressor Therapy

**Figure 2:**
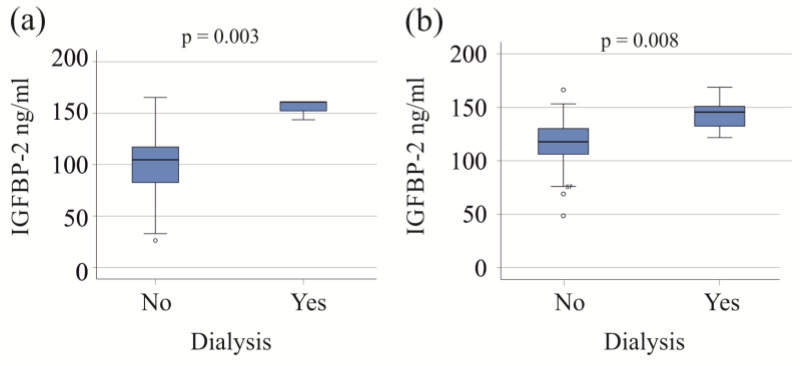
IGFBP-2 serum levels of patients with and without dialysis. (**a**) IGFBP-2 serum levels of patients with moderate COVID-19 divided according to the need for dialysis; (**b**) IGFBP-2 serum levels of patients with severe COVID-19 divided according to the need for dialysis.

Patients with both moderate and severe COVID-19, who required dialysis, exhibited increased serum IGFBP-2 levels (Figure 2). Within the severe COVID-19 group, the six patients on dialysis were older (*p* = 0.027) and had higher procalcitonin levels compared to those not needing dialysis (*p* = 0.009). An increase in procalcitonin was also observed in the moderate COVID-19 group requiring dialysis (*p* = 0.025).

Patients on vasopressor therapy all had severe disease and had higher IGFBP-2 levels than those not on vasopressor support (Table 2).

These patients also had elevated levels of CRP (*p* = 0.001) and procalcitonin (*p* < 0.001) compared to patients not using vasoactive agents.

Almost all patients with severe COVID-19 required ventilation, while none in the moderate group did, making statistical comparison non-applicable.

### 3.3. Correlation of Serum IGFBP-2 Levels with Laboratory Measures and Inflammation Markers

In moderate COVID-19 patients, IGFBP-2 levels were positively correlated with CRP, procalcitonin, LDH, AP, and IL-6, and negatively correlated with lymphocyte count. In severe COVID-19 cases, positive associations were found between serum IGFBP-2 and procalcitonin, AP, and ferritin (Table 3).

### 3.4. Effect of Bacterial Infections on Serum IGFBP-2 Levels

Critically ill patients hospitalized with severe COVID-19 are at increased risk of secondary bacterial infections [40]. Biomarkers for the early detection of bacterial infections are still lacking [40], so we analyzed whether IGFBP-2 could differentiate between these groups.

**Figure 3:**
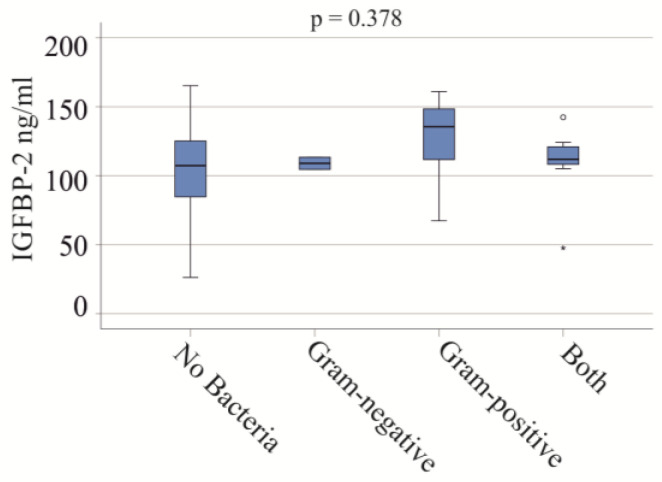
IGFBP-2 serum levels and bacterial infections of patients with severe COVID-19. Serum IGFBP-2 levels of patients not infected with bacteria, and of patients with Gram-negative bacteria, Gram-positive bacteria, and both types of bacteria.

In the severe COVID-19 group, blood infections included Gram-negative bacteria in two patients, Gram-positive bacteria in eight patients, and both types in seven patients. No significant differences in IGFBP-2 serum levels were observed between these groups (*p* = 0.378) (Figure 3). In the moderate COVID-19 cohort, one patient had a Gram-positive bacterial infection, and another had both types of bacterial infections.

It should be noted that the CRP (*p* = 0.832) and procalcitonin (*p* = 0.767) of patients with and without bacterial infections were similar.

### 3.5. Serum IGFBP-2 Levels and Survival

**Figure 4:**
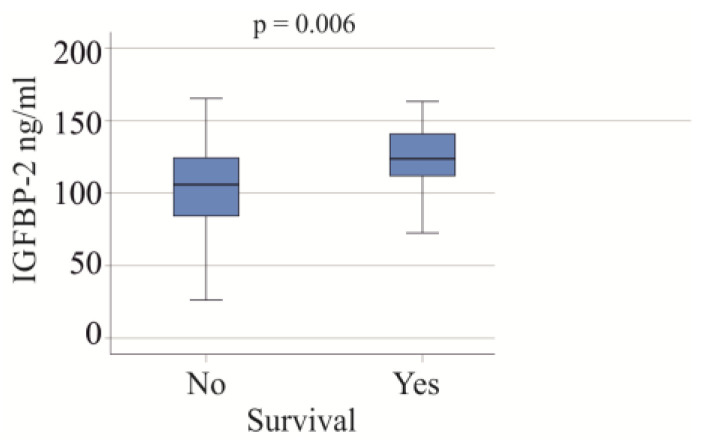
IGFBP-2 serum levels of survivors and non-survivors in the cohort with severe COVID-19.

In the group of patients with severe COVID-19, 22 individuals died, and all causes of death were attributed to COVID-19. The serum IGFBP-2 levels were found to be higher in the non-survivors (*p* = 0.006; Figure 4). After excluding patients with liver cirrhosis and adiposity, the association of serum IGFBP-2 and survival was not significant (*p* = 0.096).

## 4. Discussion

This study shows that serum IGFBP-2 is a prognostic factor for COVID-19 severity.

We found that serum IGFBP-2 levels were higher in severe COVID-19 patients compared to both healthy controls and those with moderate disease, suggesting a relationship with disease severity. Previous studies have reported increased serum IGFBP-2 levels in critical illness [4,5,41], and higher IGFBP-2 levels in severe COVID-19 are consistent with these findings.

Adiposity is a risk factor for COVID-19 severity and mortality [42]. In our cohort, 27% of patients with severe COVID-19 and 4% of patients with moderate COVID-19 were obese. Due to the relatively small number of obese patients, associations of obesity with mortality were not calculated. Most studies found an inverse relation between serum IGFBP-2 levels and body mass index [43,44,45]. Obese COVID-19 patients had lower levels of IGFBP-2 than non-obese patients. This shows that the decrease in serum IGFBP-2 in obesity that has been observed in other cohorts [43,44,45] is also present in COVID-19 patients. Thus, obesity is a confounding factor for the association of serum IGFBP-2 with markers of COVID-19 disease severity.

Male sex is a risk factor for COVID-19 severity and mortality [46]. However, the serum IGFBP-2 levels of male and female patients with COVID-19 were similar. In the control group, women had higher serum IGFBP-2 levels than men. This is in contrast to a study in the normal population that described slightly higher IGFBP-2 levels in men [47]. Estrogen has been shown to cause a decrease of IGFBP-2 in postmenopausal women [17], and differences in the hormonal status of the women between these studies may contribute to these conflicting observations, which require further investigation.

Procalcitonin and CRP are clinical markers of inflammation [48,49], and both were positively correlated with IGFBP-2. In patients with moderate and severe disease, serum IGFBP-2 levels correlated positively with procalcitonin. Positive associations with CRP were also observed in patients with moderate disease. In addition, the current analysis showed that patients requiring dialysis or vasopressor therapy had higher serum IGFBP-2 levels. All these observations suggest that serum IGFBP-2 levels have prognostic value in predicting severe COVID-19 disease.

Patients with severe COVID-19 had higher numbers of neutrophils, monocytes, and immature granulocytes in their blood compared to those with moderate disease, in line with recent findings [50]. However, lymphocyte counts were similar in both groups of patients. Given the association of lymphopenia with COVID-19 severity and higher mortality [51], particularly in moderate and severe cases, the negative correlation of IGFBP-2 with lymphocyte count in moderate COVID-19 may indicate its association with disease severity.

Patients on dialysis, all experiencing acute kidney failure due to sepsis, had higher serum IGFBP-2 levels compared to those not undergoing dialysis. This study found a positive correlation between serum IGFBP-2 and age in dialysis patients, consistent with previous findings [52]. Elevated IGFBP-2 levels in dialysis patients could partly be attributed to older age [43]. Additionally, the patients on dialysis exhibited higher procalcitonin levels, which positively correlated with serum IGFBP-2 in our study. While higher IGFBP-2 levels in patients with kidney disease have been reported, this study—for the first time—analyzes IGFBP-2 in renal failure related to sepsis.

It should be noted that serum IGFBP-2 in the controls did not correlate with age, in accordance with previous findings in a normal population [47].

The co-infection of COVID-19 patients with bacteria was observed in about 40% of our patients with severe COVID-19 and 4% of our patients with moderate disease. One meta-analysis reported a 56% co-infection rate of hospitalized COVID-19 patients [53]. A separate meta-analysis found a 5.6% prevalence of bacterial co-infections in COVID-19 [54]. The co-infection rates reported by individual studies vary widely, and our results are in this range [54]. Endotoxin, a component of the cell wall of Gram-negative bacteria, increased IGFBP-2 in the blood of healthy controls [13]. In our patient cohort, bacterial co-infection was not related to a change in serum IGFBP-2 levels. The use of endotoxin, bacterial infection, as well as SARS-CoV-2 infection induce systemic inflammation [13,19,48,49], and this can lead to higher levels of IGFBP-2 in the serum. Notably, CRP and procalcitonin levels were similar in patients with and without bacterial infections. Biomarkers for the early detection of bacterial infections are urgently needed and will contribute to improved therapeutic performance [40]. From the current analysis, we conclude that serum IGBFBP-2 is not suitable for the diagnosis of bacterial co-infection. This is also true for CRP and procalcitonin, which do not increase further in COVID-19 patients with bacterial co-infection.

In our cohort, 22 patients with severe COVID-19 died, and the cause of death in this cohort was severe COVID-19 disease. These non-survivors exhibited increased serum IGFBP-2 levels. High IGFBP-2 levels have been shown to be predictive of mortality in participants in the Baltimore Longitudinal Study of Aging [43]. The Health, Aging and Body Composition study also showed that all-cause mortality was associated with increased IGFBP-2 [44]. Plasma IGFBP-2 was associated with cardiovascular mortality in acute and chronic heart failure patients [55], and serum IGFBP-2 levels predicted mortality in patients with severe aortic stenosis [56]. Thus, higher circulating levels of IGFBP-2 are related to mortality in the general population, in patients with cardiovascular diseases [43,44,55,56], and, according to our study, in patients with COVID-19. The association between serum IGFBP-2 levels and survival was not significant after excluding patients with obesity and patients with cirrhosis. This may be related to the smaller cohort size and needs to be confirmed in larger patient groups.

IGFBP-2 exerts IGF1-dependent and -independent functions [2]. IGF1 was found to be low in the serum of patients with severe COVID-19 [30], and the present analysis showed that this is in parallel with a rise in serum IGFBP-2 levels. Increased IGFBP-2 may further impair IGF bioactivity and contribute to the hypercatabolic state of severely ill patients [57].

This observational study cannot identify the mechanisms contributing to higher serum IGFBP-2 in severe COVID-19. IGF1 is low in the serum of patients with severe COVID-19 [30], and has no effect on increased IGFBP-2. Inflammatory factors such as tumor necrosis factor may induce IGFBP-2, but this needs further study.

There are several limitations to this study. Blood samples from some patients were taken relatively late in the patients’ hospital stay. The time from the onset of COVID-19 symptoms to clinical cure ranges from 8 to 53 days, with large inter-individual variability [58], and IGFBP-2, CRP, procalcitonin, and IL-6 were not related to the time of blood collection in our cohort. Our finding of higher IGFBP-2 in severe COVID-9 does not appear to be significantly influenced by the few patients whose blood was collected at a late time point. Serum IGFBP-2 levels in severely ill patients did not decline during 30 days of follow-up, suggesting that IGFBP-2 changes little during the course of the disease [4]. A further limitation is that the BMI of the control cohort was not documented. The vaccination status of our patients against SARS-CoV-2 was not recorded. Vaccination started in Germany on 26 December 2020, suggesting that most of our patients were not vaccinated. Vaccinated people had less severe SARS-CoV-2 disease than those who were not vaccinated [59]. However, it is unlikely that the vaccination itself will change the serum levels of IGFBP-2.

## 5. Conclusions

In summary, this study shows that IGFBP-2 serum levels are a clinically relevant prognostic factor for a severe course of COVID-19 disease. Higher serum IGFBP-2 levels are associated with increased disease severity and lower survival rates in COVID-19 patients. The similarity in serum IGFBP-2 levels between patients with moderate COVID-19 and healthy controls indicates that elevated IGFBP-2 is linked to critical illness rather than SARS-CoV-2 infection itself. Therefore, elevated systemic IGFBP-2 is a marker of severe illness and may be useful in monitoring disease severity and guiding therapy in COVID-19 patients.

## Figures and Tables

**Table 1:** Characteristics of the study cohort. Data of COVID-19 patients with moderate and severe disease are listed. Data are given as median values, and the numbers in brackets are the minimum and the maximum values. In case laboratory values were not recorded for all patients, the number of patients for whom these data were available is given in superscript. The *p*-values are * *p* < 0.05, ** *p* < 0.01, and *** *p* < 0.001.

Parameter	Moderate COVID-19	Severe COVID-19
Males/females	32/25	42/18
BMI kg/m^2^	26.2 (18.4–42.6)^25^	29.4 (19.2–66.7)^56^ **
Age (years)	58 (22–83)	57 (31–83)
C-reactive protein mg/L	20 (0–218)^53^	74 (1–367) ***
Procalcitonin ng/mL	0.09 (0–25)^32^	0.24 (0–25) **
LDH U/L	215 (82–929)^47^	378 (162–1534) ***
AP U/L	84 (38–372)^39^	99 (37–743)
Ferritin ng/mL	601 (68–4237)^27^	1088 (77–21976) *
IL-6 pg/mL	30 (4–265)^22^	36 (3–1175)
Neutrophils n/nL	4.05 (0.13–12.16)^51^	8.18 (0.90–24.91) ***
Eosinophils n/nL	0.07 (0–0.88)^51^	0.05 (0–1.07)
Monocytes n/nL	0.53 (0.07–1.49)^51^	0.71 (0.03–2.21) **
Lymphocytes n/nL	1.08 (0.12–57.83)^51^	1.20 (0–75.95)
Immature granulocytes n/nL	0.03 (0–0.78)^51^	0.25 (0.04–2.92) ***

**Table 2:** Comparison of serum IGFBP-2 levels in patients with and without dialysis, with and without ventilation, and with and without vasopressor therapy. The number of patients treated is given in the “N” columns, and the corresponding *p*-values are listed.

Intervention/Drug	Moderate COVID-19		Severe COVID-19	
	N	*p*-Value	N	*p*-Value
Dialysis	3	0.003	6	0.008
Ventilation	0	Not applicable	41	Not applicable
Vasopressor therapy	0	Not applicable	28	0.025

**Table 3:** Spearman correlation coefficients (r) and *p*-values for the correlations of serum IGFBP-2 with laboratory values in moderate and severe COVID-19 patients (alkaline phosphatase, AP; interleukin-6, IL-6; lactate dehydrogenase, LDH).

Laboratory Measures	Moderate COVID-19		Severe COVID-19	
	r	*p*-Value	r	*p*-Value
C-reactive protein mg/L	0.508	<0.001	0.266	0.084
Procalcitonin ng/mL	0.660	<0.001	0.533	<0.001
LDH U/L	0.320	0.041	0.126	0.422
AP U/L	0.443	0.010	0.313	0.041
Ferritin ng/mL	0.379	0.081	0.397	0.008
IL-6 pg/mL	0.458	0.049	0.053	0.737
Neutrophils n/nL	0.148	0.330	0.068	0.665
Eosinophils n/nL	0.141	0.354	0.087	0.579
Monocytes n/nL	−0.057	0.712	−0.152	0.332
Lymphocytes n/nL	−0.484	0.001	−0.034	0.831
Immature granulocytes n/nL	0.277	0.066	−0.088	0.575

## Data Availability

Data supporting the reported results can be obtained from the corresponding author.
